# A Dissipation Function–Based Method for Calculating the Energy Loss of Intracranial Aneurysms

**DOI:** 10.3389/fneur.2021.639690

**Published:** 2021-07-08

**Authors:** Xiao Mo, Hongshi Yu, Rong Chen, Zhenpeng Chen, Haiyun Li

**Affiliations:** ^1^Beijing Key Laboratory of Fundamental Research on Biomechanics in Clinical Application, School of Biomedical Engineering, Capital Medical University, Beijing, China; ^2^School of Energy and Power Engineering, Xi'an Jiaotong University, Shanxi, China

**Keywords:** intracranial aneurysms, hemodynamics, energy loss, dissipation function, computational fluid dynamics

## Abstract

At present, the energy loss (EL) mechanism of intracranial aneurysm (IA) rupture is explored based on the global EL calculated by Bernoulli equation, but the details of EL are still unclear. This study aimed to explore the temporal and spatial characteristics of EL of IAs and reveal its mechanism. A novel method for calculating the EL of IAs based on dissipation function (DF) was proposed. DF was derived from the differential form of the energy equation and reflected the irreversible conversion from mechanical energy to internal energy caused by the friction between the fluid micelles. Eight sidewall IAs located at the posterior communicating segment of the internal carotid artery were collected; the three-dimensional (3D) geometric models of IAs were established employing image segmentation and 3D reconstruction. Computational fluid dynamics was applied to obtain hemodynamic parameters of IAs. The temporal and spatial characteristics of EL of IAs were achieved utilizing our proposed method. The simulation results indicated that EL occurred mainly in the boundary layer and the region adjacent to high-velocity inflow jet, EL increased rapidly during cardiac systole and reached its maximum at end-systolic phase and then decreased gradually during diastole until the end of cardiac cycle. The proposed method achieved some improvements over the traditional Bernoulli equation–based method by acquiring the temporal and spatial characteristics of EL, and it could provide insights into the EL of IAs and contribute to further rupture mechanism investigation.

## Introduction

Intracranial aneurysms (IAs) are cerebrovascular pathology that mostly occurs near the circle of Willis (1, 2). Their rupture would frequently cause subarachnoid hemorrhage, with high mortality and long-term disability (3–5). The hemodynamic mechanism of IA rupture has attracted considerable attention. Studies have shown that hemodynamic environment plays an important role in the growth and rupture of IAs, and many hemodynamic parameters associated with the risk of IA rupture have been identified, such as wall shear stress, oscillatory shear index, gradient oscillatory number, oscillatory velocity index, fluctuating kinetic energy, and energy loss (EL). Intuitively, the process of IA rupturing involves energy absorption. There is a certain correlation between EL of IAs and their rupture risk; in particular, EL is likely to contribute to IA clinical rupture risk stratification. EL was defined as irreversible conversion from mechanical energy to internal energy due to the viscous dissipation of fluid. Qian et al. (6) first found that EL of ruptured IAs was significantly higher than that of unruptured IAs, and EL exhibited a better ability to predict the risk of IA rupture compared to time-average wall shear stress. Ruptured IAs tended to have more complex flow patterns and a longer circulation track of blood flow in the aneurysm sac. Through multivariate logistic regression analysis, Liu et al. (7) considered that EL was an independent significant parameter to evaluate the rupture risk of IAs. Moreover, for every 10% increase in EL, the probability of IA rupture increased by 1.65 times. Similarly, Qin et al. (8) also found that EL in ruptured group was significantly higher than that in the unruptured group. Multivariate regression analysis suggested that EL was one of the significant predictors of the rupture of middle cerebral artery bifurcation aneurysms. Farnoush et al. (9) focused on the rupture risk of bifurcation IAs during clinical patient follow-up. They found that IAs with increasing EL during follow-up were more likely to rupture than those with decreasing EL. Long et al. (10) established an aneurysm scaling model to explore the relationship between hemodynamic characteristics and aspect ratio. Their results showed that EL elevated with increasing aspect ratio. Moreover, higher rupture risk might be associated with sharply increasing EL during IA growth. However, Takao et al. (11) asserted that although EL was higher in 13 ruptured aneurysms than that in 87 unruptured aneurysms, no significant difference was observed between the two groups. The same conclusion was reached in a study focusing on internal carotid artery aneurysms (12). Clearly, there are contradictions in the existed findings. Whether EL is recognized as a risk factor for IA rupture remains to be further explored. All the existing studies applied Bernoulli equation to obtain the global EL of IAs. Applying global EL may make it difficult to distinguish between ruptured and unruptured IAs. In this article, we proposed a method based on the dissipation function (DF) to calculate the EL of IAs, which could illustrate the temporal and spatial characteristics of EL and reveal its change mechanism. This method provides a new way to explore the EL within IAs and has advantages over Bernoulli equation–based method in exploring the EL mechanism of IA rupture.

## Methods

### Image Data and the IA Models

**Figure 1:**
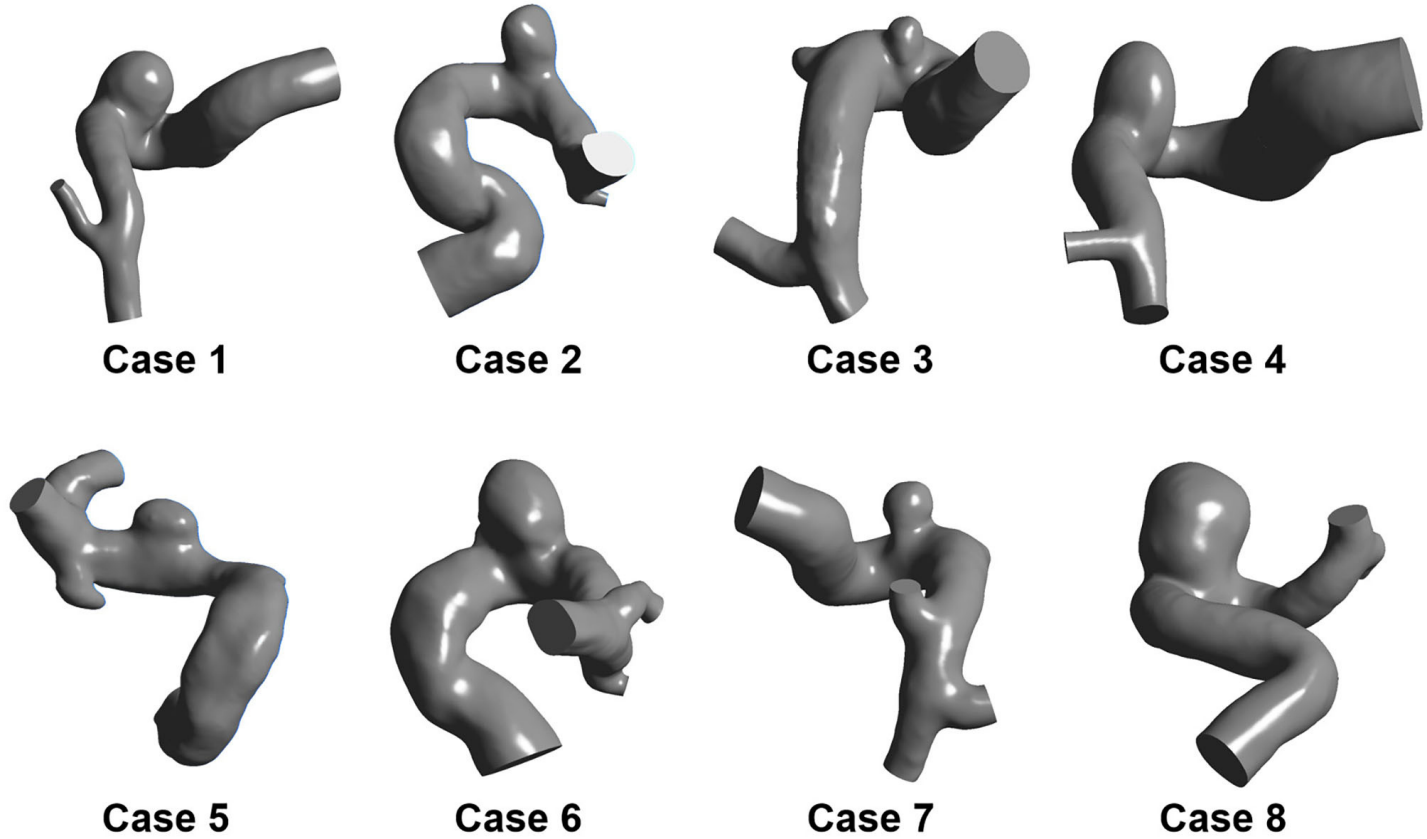
Eight patient-specific IA models.

Eight sidewall IAs located at the posterior communicating segment of the internal carotid artery were collected from Beijing Tiantan Hospital Affiliated to Capital Medical University. All the participants provided their written informed consent. Besides, the protocol of this study was approved by the Ethics Committee of Beijing Tiantan Hospital Affiliated to Capital Medical University. The three-dimensional (3D) rotational angiography (3DRA) images of these patients were captured using a GE LCV + Digital Subtraction system (LCV; GE Medical Systems) during rotation of 200° at a rate of 8.8 frames per second. Then, the 88 projection images were transmitted to a 3D dataset using isotropic voxels on a dedicated GE workstation (Advantage Unix; GE Medical Systems). Afterward, the acquired raw DICOM files were inputted into the proprietary software Mimics 10.0 (Materialize, Leuven, Belgium). Next, the IA geometry was extracted and then converted into a triangulated surface model based on a manually set image cropping threshold. Subsequently, the surface along the normal direction of the wall was extracted to build the blood vessel wall with the software Geomagic Studio 12 (Raindrop Geomagic, Durham, NC, USA). Finally, the established model was modified as a 3D-solid volume model with software SolidWorks 2012 (SolidWorks Corp., Concord, MA, USA). Accordingly, the eight IA models were formed (Figure 1).

### Numerical Simulation

The hemodynamic characteristics were acquired using computational fluid dynamics (CFD). The computational meshes were generated by ANSYS ICEM 18.0. Mesh independence studies were conducted, and the element size was finally set to 0.13 mm. The seven boundary-fitted prism layers were generated with an average node space increasing by a ratio of 1.3. Besides, the flow-governing Navier–Stokes equation was solved using ANSYS Fluent 18.0. The aneurysm wall was assumed to be no-slip and rigid. Afterward, the blood was modeled as an incompressible under laminar flow conditions, of which the density and dynamic viscosity were set to 1,060 kg/m^3^ and 0.004 N·s/m^2^, respectively. The inlet boundary conditions included a pulsatile velocity that was measured through a transcranial Doppler examination. At the outlets, a zero pressure gradient was applied (10). The cardiac cycle was 0.8 s with a time step of 0.01 s for each cardiac cycle. A total of 3 cycles were simulated while only the last one was chosen for output. Then, the hemodynamic parameters were computed using ANSYS CFD-Post.

**Figure 2:**
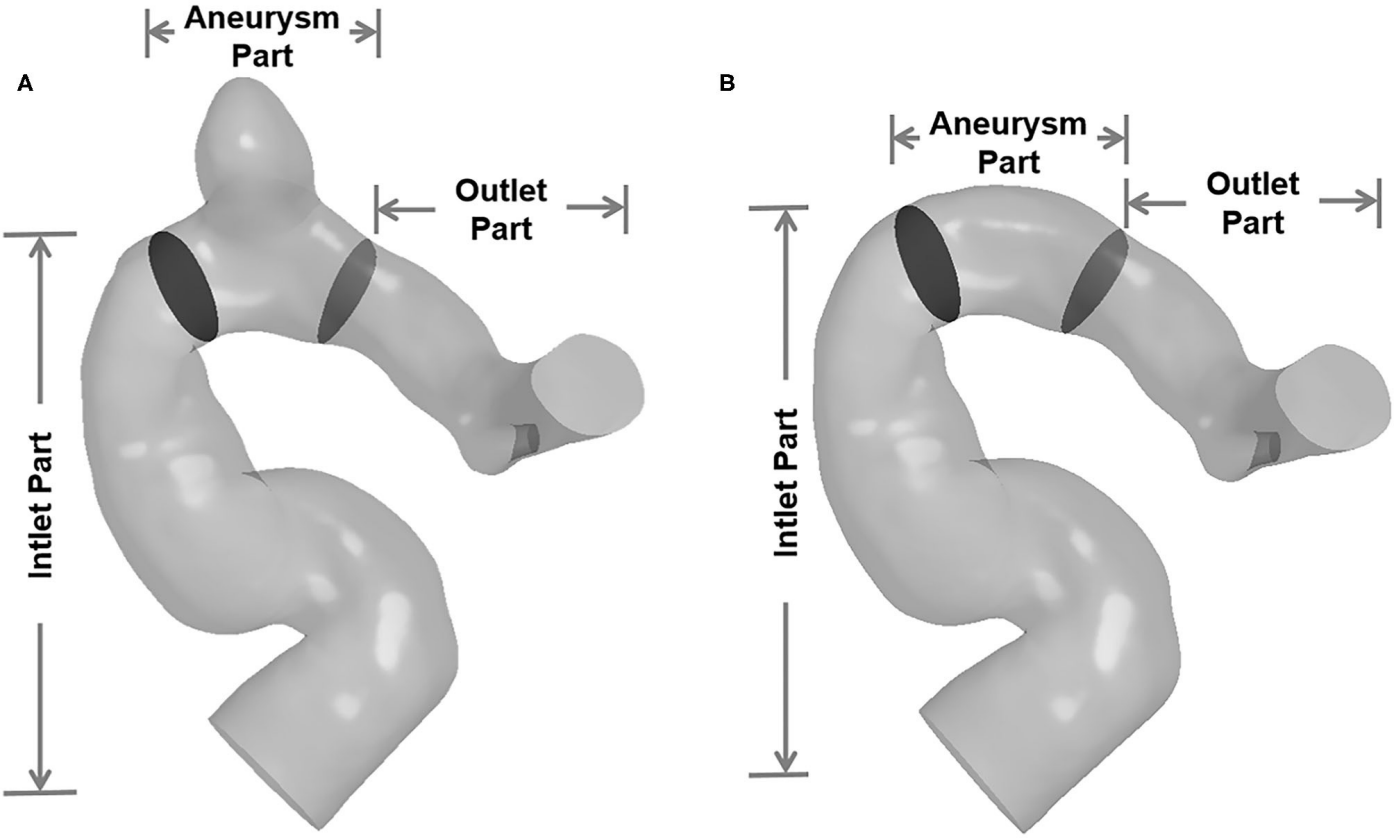
The IA models were divided into three segments (inlet part, aneurysm part, and outlet part). **(A)** Three segments of with-aneurysm model. **(B)** Three segments of non-aneurysm model.

In order to facilitate revealing the spatial characteristics of EL of IAs, the IA models were divided into three segments: inlet part, aneurysm part, and outlet part (Figure 2). In addition, the ELs of “with-aneurysm” and “nonaneurysm” models [refer to Qian et al. (6)] were adopted to explore the EL changes caused by the aneurysm formation.

### DF-Based EL Computing

In general, EL is defined as the irreversible conversion from mechanical energy to internal energy caused by the friction between the fluid micelles. It was commonly calculated by the Bernoulli equation in previous studies. This method mainly focused on the calculation of the overall EL of the entire fluid in a segment of the blood vessel with IAs. We proposed a DF-based EL computing method to explore the temporal and spatial characteristics of EL within IAs. DF is derived from the differential form of energy equation.

The energy equation can be expressed as follows (13):

(1) $$\begin{array}{l} \frac{\partial \left(\rho h\right)}{\partial t}+\frac{\partial \left(\rho uh\right)}{\partial x}+\frac{\partial \left(\rho \vartheta h\right)}{\partial y}+\frac{\partial \left(\rho wh\right)}{\partial z}=-P\left(\nabla •\vec{V}\right)\\ \;+\nabla •\left(\lambda \nabla T\right)+\Phi +S_{h} \end{array}$$

where ρ is the density; *h* is the enthalpy; *t* is the time; and *u, v*, and *w* are the velocity components in *x, y*, and *z* directions, respectively. *T* is the temperature, *P* is the pressure, λ is the thermal conductivity, *S*_h_ is the internal heat source of fluid, and Φ is defined as DF that refers to the part of internal energy converting from mechanical energy due to viscous dissipation. DF is expressed as follows:

(2) $$\begin{array}{l} \text{DF}=2\mu \left[{\left(\frac{\partial u}{\partial x}\right)}^{2}+{\left(\frac{\partial v}{\partial y}\right)}^{2}+{\left(\frac{\partial w}{\partial z}\right)}^{2}\right]\\ \;+\mu \left[{\left(\frac{\partial v}{\partial x}+\frac{\partial u}{y}\right)}^{2}+{\left(\frac{\partial w}{\partial y}+\frac{\partial v}{\partial z}\right)}^{2}+{\left(\frac{\partial u}{\partial z}+\frac{\partial w}{\partial x}\right)}^{2}\right] \end{array}$$

where μ is the dynamic viscosity. In physics, DF is the work done by surface forces in the linear deformation and shear deformation process of fluid micelles. This is also the mechanism of EL generation. The dimension of the DF is W/m^3^, which indicates the viscous dissipation power (DP) per unit volume. Given this, DF distribution can exhibit the EL distribution.

Any enclosed fluid domain in the blood vessel can be chosen as control volume (CV), and the DP can be obtained by integrating DF over CV:

(3) $$\begin{array}{l} \text{DP}=\underset{\text{CV}}{∭}\text{DFdv} \end{array}$$

DP is the transient EL in the CV at a certain moment.

The total EL of a cardiac circle, EL_df_, was defined as the integral of DP over a cardiac cycle:

(4) $$\begin{array}{l} \text{E}{\text{L}}_{\text{df}}=\frac{\int_{t_{0}}^{t_{0}+n\Delta t}\text{DPdt}}{n} \end{array}$$

where Δ*t* refers to the cardiac cycle, *t*_0_ represents the integral start time, and *n* denotes the number of cardiac cycles.

In the existed studies, EL was calculated by Bernoulli equation–based method. Here it is denoted as EL_be_; EL_be_ is actually the total EL of a cardiac circle. The expression of EL_be_ is as follows:

(5) $$\begin{array}{l} \text{E}{\text{L}}_{\text{be}}=\frac{\int_{t_{0}}^{t_{0}+n\Delta t}ṁ\left[\left(\frac{1}{2}V_{\text{in}}^{2}-\frac{1}{2}V_{\text{out}}^{2}\right)+\left(\frac{P_{\text{in}}}{\rho }-\frac{P_{\text{out}}}{\rho }\right)\right]\text{dt}}{n} \end{array}$$

where ṁ denotes the mass flow rate; *P* represents the pressure; *V* refers to the velocity; and *in* and *out* indicate inlet and outlet of CV, respectively.

## Results

Applying our proposed method on the eight patient-specific IA models, the spatial and temporal characteristics of EL were achieved.

### The Spatial Characteristics of EL

**Figure 3:**
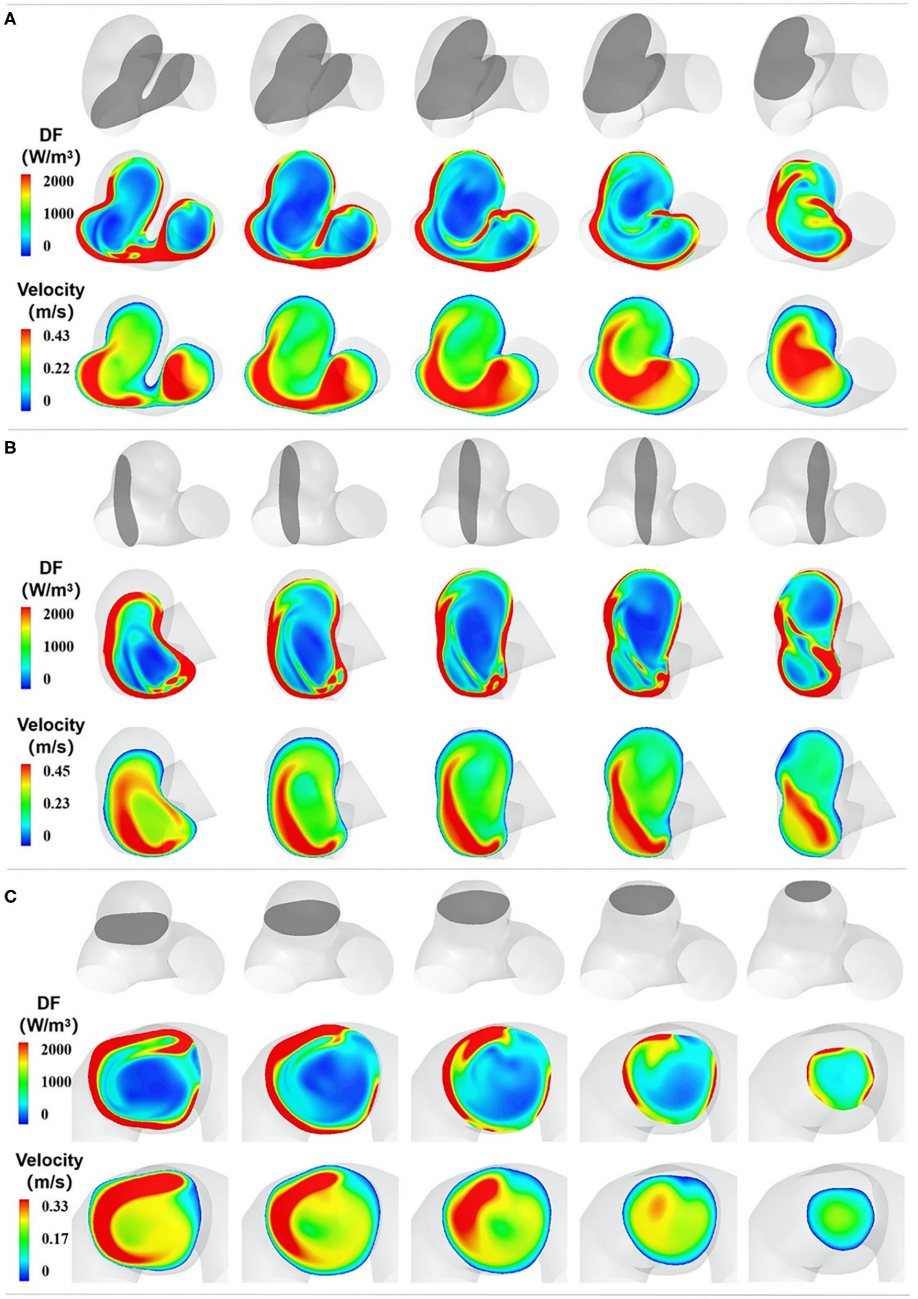
The spatial characteristics of EL. **(A–C)** represent the DF distribution of three orthogonal cutting planes, respectively. In each section, the top row indicates the cutting plane, which is colored gray. The second row exhibits the DF distribution of the chosen plane, and the third row shows the velocity distribution of the chosen plane.

We adopted the DF of three orthogonal cutting planes to exhibit the spatial characteristics of EL (Figure 3). The first cutting planes (denoted as C) were paralleled to the aneurysm neck plane, the second cutting plane (denoted as B) was perpendicular to the flow direction, and the third plane was denoted as A. The spatial characteristics of EL illustrated that high-DF regions were consistent with high-velocity-gradient regions, including boundary layers and the regions adjacent to high-velocity inflow jet. DF gradually decreased with the distance from the vessel wall, and the low-DF regions were mainly located in the center of the aneurysm sac.

### The Temporal Characteristics of EL

**Figure 4:**
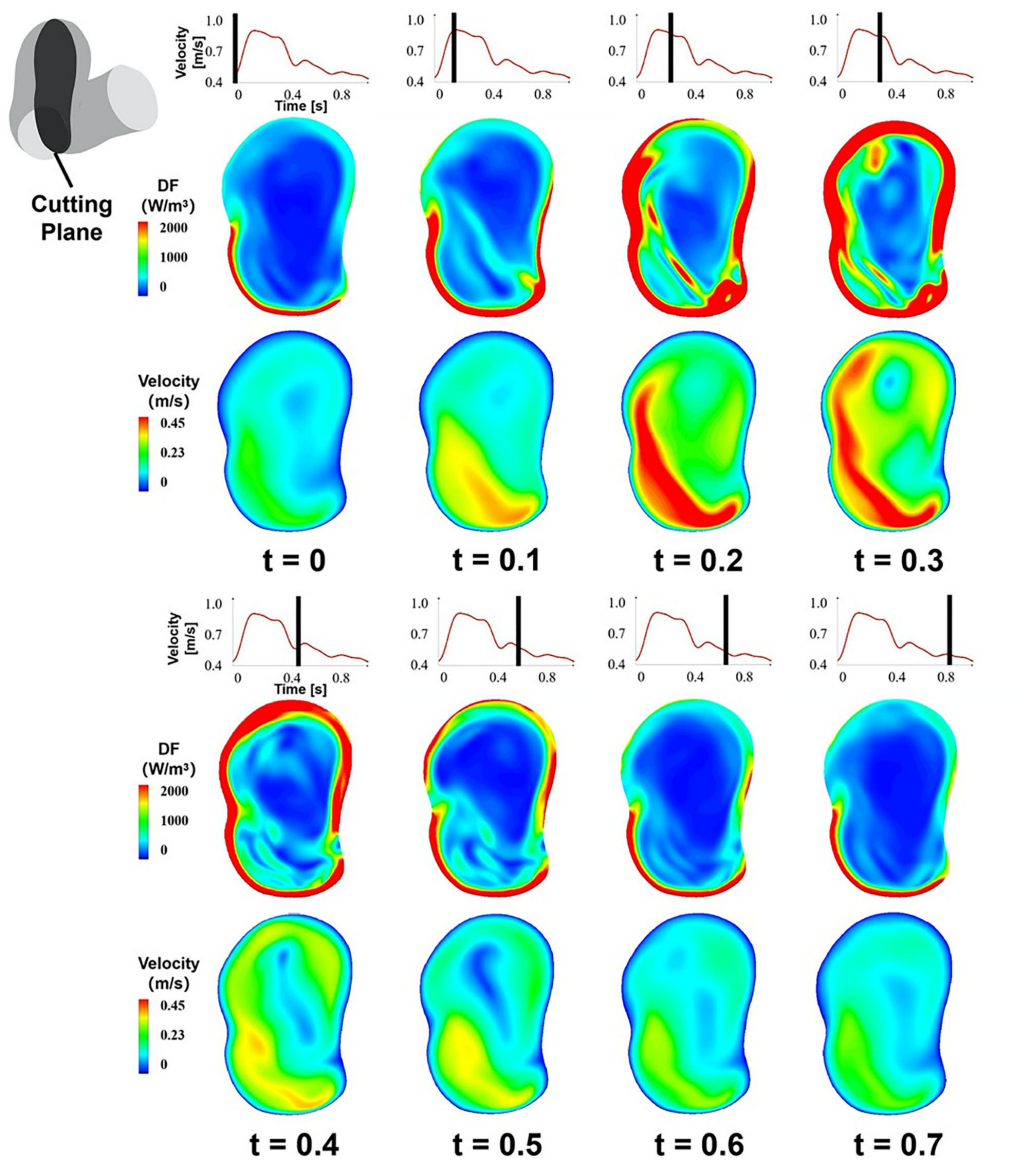
The temporal characteristics of EL, the DF of the cutting plane B during a cardiac circle.

We adopted the DF of the cutting plane B during a cardiac circle to exhibit the temporal characteristics of EL (Figure 4). The temporal characteristics of EL illustrated that with the increase in the inlet velocity, the high-DF area expanded, and the maximum value of DF in aneurysm sac also elevated. By contrast, the high-DF area shrunk as the inlet velocity decreased.

**Figure 5:**
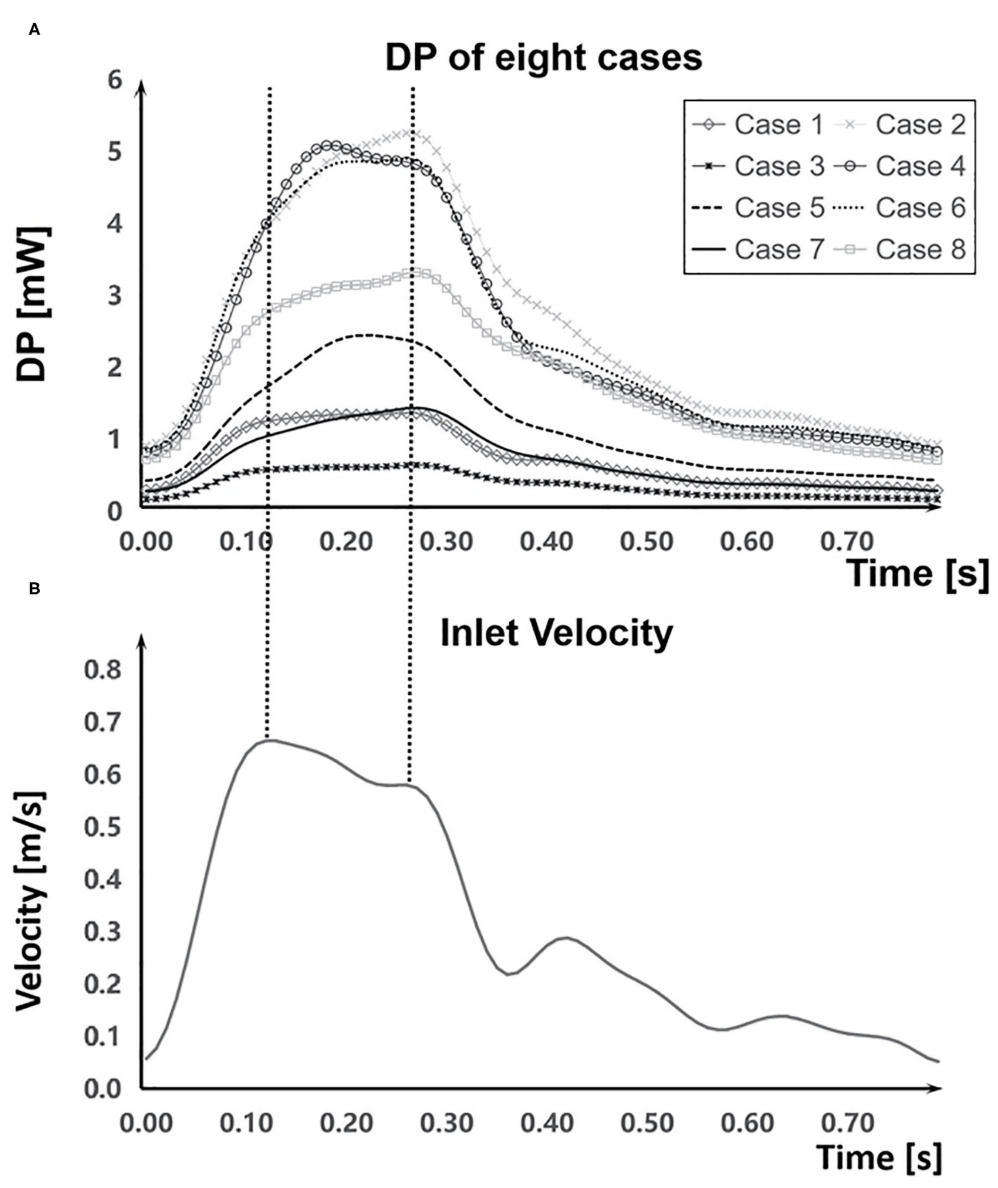
Comparation of DP and the inlet velocity during a cardiac cycle. **(A)** DP in aneurysm part during a cardiac cycle of each case. **(B)** The inlet velocity of inlet part.

The DP in aneurysm part and the inlet velocity magnitude of the inlet part during a cardiac cycle were obtained (Figures 5A,B). The inlet velocity and DP had similar temporal characteristics. EL first increased during the systolic period, reaching the maximum at the end of the systolic period, and then decreased during the diastolic period, reaching the minimum at the end of the cardiac cycle. Besides, DP had a certain degree of hysteresis because it needed some time for blood to flow into the aneurysm part from the inlet part.

### Main Effect Factors

**Figure 6:**
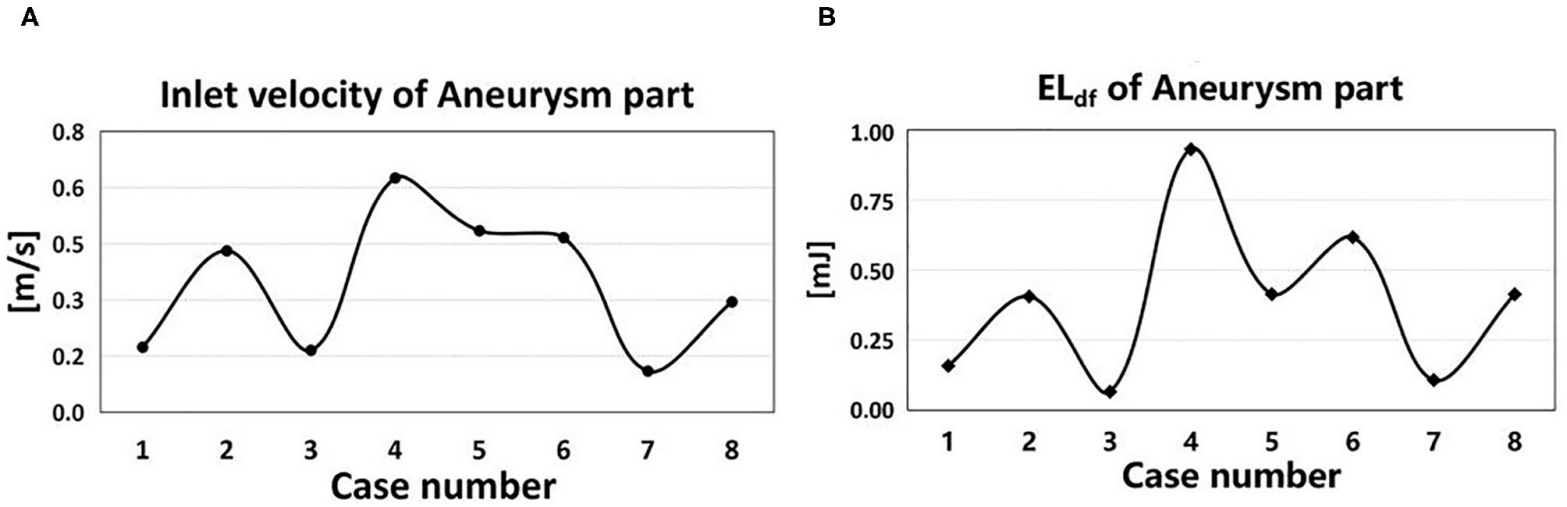
Inlet velocity and EL of eight cases. **(A)** The time-averaged velocity magnitude at the inlet of aneurysm part in eight cases. **(B)** The EL_df_ of aneurysm part in eight cases.

The EL mainly depended on the inlet velocity and the morphological characteristics of IAs. EL had a positive correlation with the inlet velocity (Figure 6). EL_df_ in the aneurysm part of the eight cases showed a strong positive correlation with the velocity magnitude (*r* = 0.928, DP increased 11.4% per 10% velocity increased), indicating that the inlet velocity magnitude of aneurysm part was tightly related to EL_df_.

**Figure 7:**
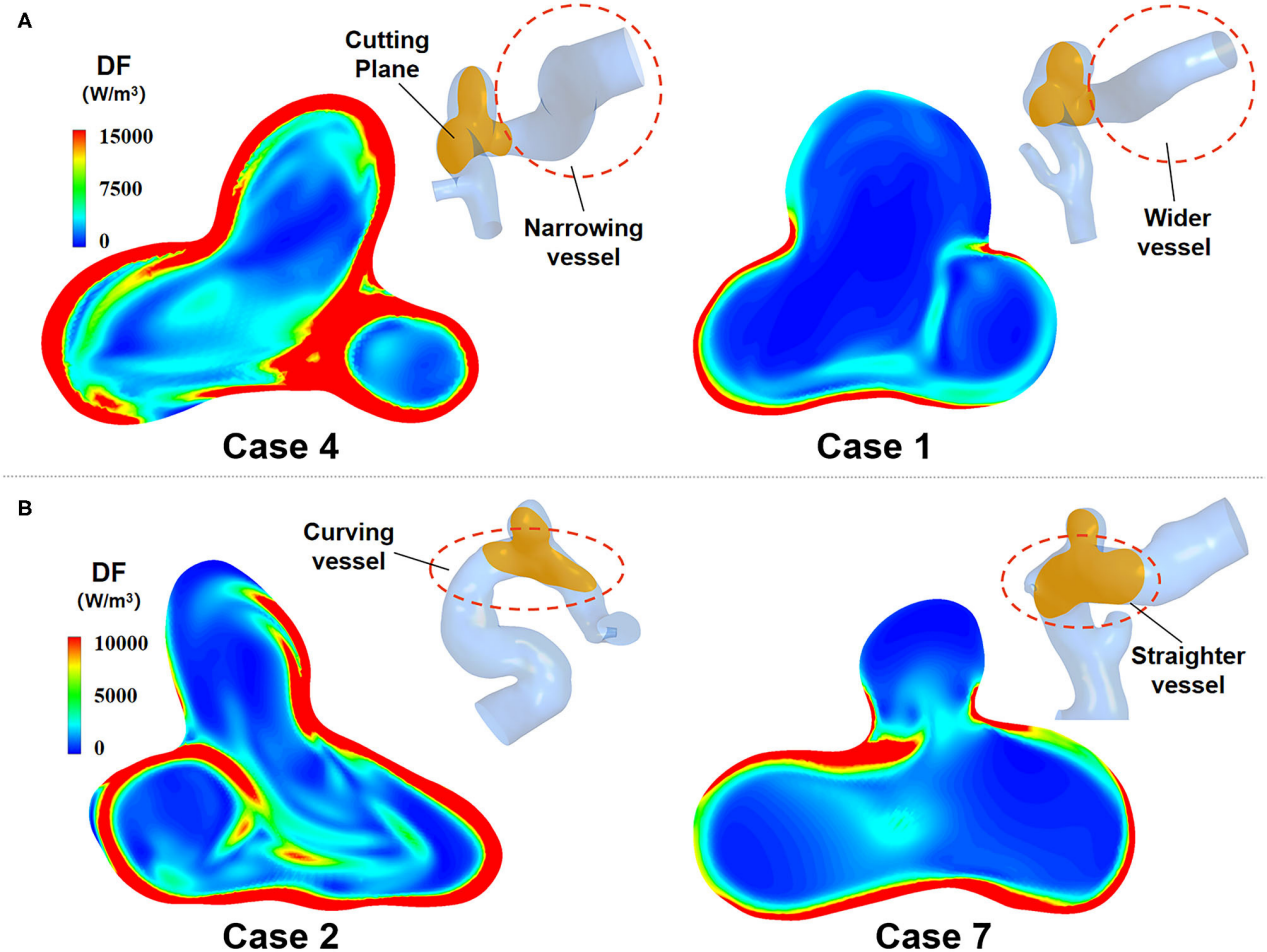
EL of IAs with two different morphological characteristics. **(A)** Narrowing parent vessel has obviously higher DF. **(B)** Curving parent vessel upstream of aneurysm sac has higher DF.

The morphological characteristics of IAs directly affected the distribution of inlet velocity and thus had an impact on the EL (Figure 7). Experiment results showed that the narrowing or curving parent vessel upstream of aneurysm sac resulted in an obviously higher DF compared to a relatively wider or straighter parent vessel.

### The EL Value Calculated by the Two Methods

**Figure 8:**
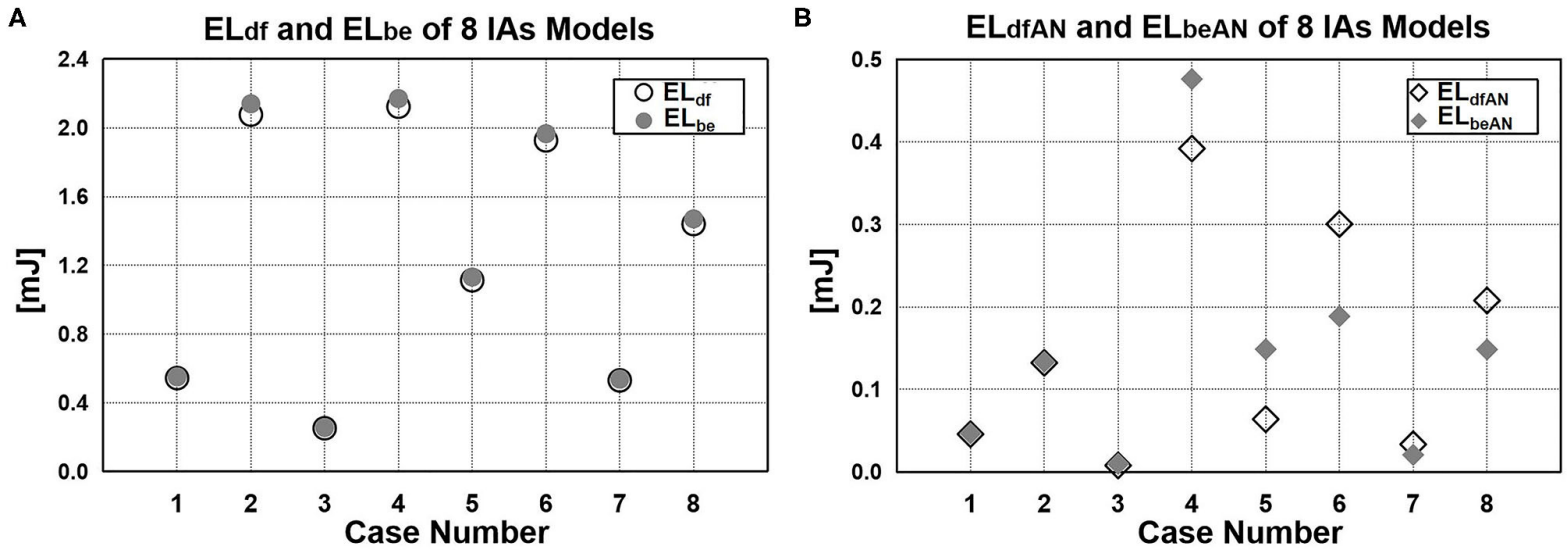
EL value calculated by DF-based and Bernoulli equation–based method. **(A)** Difference between EL_df_ and EL_be_. **(B)** EL of aneurysm sac: EL_dfAN_ and EL_beAN_.

We employed DF-based and Bernoulli equation–based method to calculate the EL of the eight cases. Figure 8A shows that the EL_df_ and EL_be_ of all three segments (inlet part, aneurysm part, and outlet part) in each case. We found that EL_be_ was slightly bigger than EL_df_. There was only 2% average difference between EL_df_ and EL_be_.

Moreover, we also compared the EL of aneurysm sac calculated by the two methods (Figure 8B). In previous studies in which the Bernoulli equation–based method was applied (6, 12, 14–17), the EL of aneurysm sac (EL_beAN_) was characterized by the EL changes caused by the aneurysm formation, namely, the difference of EL_be_ between with-aneurysm and non-aneurysm models. In our method, the EL of aneurysm sac (EL_dfAN_) can be obtained by integrating DF over aneurysmal domain. As suggested from the quantitative comparison, the difference of EL_dfAN_ and EL_beAN_ over eight cases varied considerably in the range of 0.8%−46.9%. The average difference reached 25.2%.

## Discussion

In the previous studies, the EL of IAs was usually calculated by applying Bernoulli equation. This method could only acquire the overall EL of the entire fluid in a segment of the blood vessel with IAs, lacking the detailed information of EL. We proposed a DF-based EL computing method, which could illustrate the temporal and spatial characteristics of EL and reveal its change mechanism. In order to explore the temporal and spatial characteristics of EL within IAs, eight patient-specific IA models were reconstructed from clinical IAs 3DRA image data, and CFD simulations were conducted. The simulation results might give us some insight into the EL within IAs. Compared with Bernoulli equation–based method, the proposed method might be more helpful to explore the EL mechanism of IA rupture.

### Method Validation

For a given IA, the EL is constant. Whether DF-based method or Bernoulli equation–based method is adopted, the total EL of a cardiac circle of the same CV calculated by the two methods should be consistent. Comparing the EL_df_ and EL_be_ of three parts (inlet part, aneurysm part, and outlet part) in each case, the difference was found to be 2% on average. The very similar experimental results from the two methods demonstrated that the DF-based method was reliable to some extent.

### Method Comparison

The DF-based method and Bernoulli equation–based method both indicate the transformation from mechanical energy to internal energy caused by viscous dissipation, which are derived from the differential and integral form of the energy equation, respectively. The DF-based method focuses on the fluid micelles, whereas the Bernoulli equation–based method concerns the entire fluid in a segment of the blood vessel. The former illustrates the physical mechanism of viscous dissipation from the microscopic perspective, and the latter calculates the energy transformation caused by viscous dissipation from a macroscopic point of view. Compared with Bernoulli equation–based method, DF-based method has several advantages. On the one hand, DF-based method can offer the spatial characteristics of EL, whereas the Bernoulli equation–based method only provides the overall quantity of EL. On the other hand, DF-based method can acquire the temporal characteristics of EL, as illustrated in Figure 7, EL varied over time. However, Bernoulli equation–based method just can achieve a time-average EL over one cardiac circle, making it unable to obtain the transient characteristic of EL. In this study, the transient EL, namely DP, can be obtained by integrating the DF over CV, compensating the deficiency of the Bernoulli equation–based method.

Finally, regarding the EL of aneurysm sac, Bernoulli equation–based method indirectly obtains its value by calculating the EL difference between the vascular model with and without aneurysm, ignoring the flow field changes caused by aneurysm formation. As shown in Figure 8B, the difference between EL_dfAN_ and EL_beAN_ in the cases varies considerably in the range of 0.8–46.9%; the average difference reaches 25.2%, indicating that the EL in aneurysm sac cannot be represented by the EL changes caused by the aneurysm formation. By contrast, the DF-based method can directly obtain EL by integrating the DF over aneurysm sac; thus, this method is more reasonable and reliable.

### EL Mechanism

Equation (2) expresses the cause of EL from the microlevel. Specifically, EL is the work done by the surface forces in the linear and shear deformation process of fluid micelles and can be easily understood as friction between fluid micelles. From a macrolevel, EL can be considered to be the work done by shear stress in the flow field with non-uniform velocity distribution. Overall, the velocity gradient of a fluid is a prerequisite for its EL. This can also be seen in the expression of DF that the magnitude of DF depends on fluid viscosity and velocity gradient. If blood viscosity remains constant, the velocity gradient is the only determinant of EL. Thus, the region with a high-velocity gradient is where EL occurs. As shown in Figure 3, in addition to boundary layers, high-velocity-gradient regions are mostly located in regions adjacent to high-velocity inflow jet. When the inflow jet flows into the aneurysm sac, surrounding blood is “dragged” by the higher-velocity inflow, causing a high-velocity gradient region. Besides, the high-velocity-gradient region is consistent with the high-DF region.

In IAs, the velocity gradient distribution mainly depends on the velocity magnitude and the morphological characteristics of IAs. As exhibited in Figure 4, EL increases with the velocity magnitude during a cardiac circle; meanwhile, the velocity gradient in boundary layers and inflow jet adjacent region also elevates. From another perspective, a higher velocity magnitude is inclined to enhance the unevenness of the flow field, leading to a higher velocity gradient and EL.

As for the effect of the morphological characteristics of IAs on their EL, it can be seen from Figure 7 that narrowing or curving parent vessel upstream of aneurysm sac results in an obviously higher DF compared to a relatively wider or straighter parent vessel. Specifically, curving parent vessel would enhance the unevenness of velocity distribution at the inlet of the aneurysm part, and narrow parent vessel would increase velocity magnitude, which both result in a higher EL. Besides, the curving parent vessel directly or indirectly determines the inflow jet direction and the impingement zone, influencing the velocity distribution of the boundary layer and the quantity of the EL.

### Association of EL With IA Rupture

From a biomechanical perspective, IAs will rupture when the mechanical stress implemented on aneurysmal wall exceeds the strength of the wall. Some pathological investigations suggested that wall degenerative remodeling would weaken the aneurysmal wall strength, making it prone to rupture (18, 19). The current consensus is that unfavorable hemodynamic environment triggers wall degenerative remodeling and consequent rupture. The unfavorable hemodynamic environment may lead to higher EL. Does high EL necessarily cause IAs to rupture? Some studies suggested that higher EL might convert into more intense physical stimulus on the pathologic aneurysm surfaces and result in wall degenerative remodeling and consequent rupture (6, 7). Others found that there was no significant difference in EL between unruptured and ruptured IAs (11, 12). The ELs involved in these studies were all global ELs calculated based on Bernoulli equation. Thus, the global EL is unlikely to be used to predict the risk of IA rupture. However, the temporal and spatial characteristics of EL implicitly describe the hemodynamic environment of IAs. So, the temporal and spatial characteristics of EL rather than global EL are more correlated with IA rupture. We speculated that both the high EL and the unfavorable hemodynamic environment in the high EL area contribute to the rupture of IAs. According to the spatial characteristics of EL in aneurysm sac, if the high EL occurs in a more concentrated impingement zone, the remodeling of the vessel wall in that area will be elevated, leading to consequent rupture. This can be confirmed by Imbesi and Kerber (20) that impingement zones were consistent with rupture sites.

## Limitations

In this article, DF is applicable to Newtonian fluids. The blood vessel is regarded as a rigid wall. Considering that inlet velocity and viscosity of blood in each patient are unavailable, the inlet flow velocity of only one normal person was adopted, and the viscosity was set as a fixed value. Consequently, these assumptions would affect our quantitative CFD results to some extent.

The present study could be considered to be a preliminary probe owing to a limited sample size, and the influence of morphological parameters on EL has not been investigated in depth. Therefore, a longitudinal study should be performed for further investigation.

## Conclusion

In this article, a DF-based method to compute the EL of IAs was established, which could illustrate the temporal and spatial characteristics of EL and reveal its change mechanism. This method will provide an effective new method for calculating and analyzing the EL mechanism of IA growth and rupture.

## Data Availability Statement

The raw data supporting the conclusions of this article will be made available by the authors, without undue reservation.

## Ethics Statement

The studies involving human participants were reviewed and approved by the Ethics Committee of Beijing Tiantan Hospital Affiliated to Capital Medical University. The patients/participants provided their written informed consent to participate in this study.

## Author Contributions

XM and HY: original concept and analyzed CFD data. HL: directed the research. HY: designed CFD experiments. XM, RC, and ZC: performed CFD experiments. XM, HY, and HL: wrote the paper. HL, HY, XM, RC, and ZC: revised the paper. All authors contributed to the article and approved the submitted version.

## Conflict of Interest

The authors declare that the research was conducted in the absence of any commercial or financial relationships that could be construed as a potential conflict of interest.
